# INFLUENCES OF AGE DIVERSITY CLIMATE ON ORG PERFORMANCE: ROLE OF INCLUSION AND ORGANIZATIONAL CITIZENSHIP BEHAVIOR

**DOI:** 10.1093/geroni/igad104.3006

**Published:** 2023-12-21

**Authors:** Soondool Chung, Ahyoung Lee

**Affiliations:** Ewha Womans University, Seoul, Republic of Korea; Ewha Womans University, Seoul, Republic of Korea

## Abstract

With the rapidly aging population, South Korea’s workforce demographics are changing as more older adults remain in the workforce. With limited resources such as work opportunities and pensions, intergenerational conflict may arise in the workplace while employers seek to maximize organizational outcomes. The present study investigated the effects of age diversity climate on organizational performance in private sector employees by focusing on the mediating effects of inclusion and organizational citizenship behavior (OCB). The data for this study was collected online in January 2022 from 900 private sector employees aged 20 to 69 in South Korea. Age-diversity climate, sense of inclusion, OCB, and organizational performance were measured using standardized scales. To test our assumed relationships, we used structural equation modeling and executed bootstrapping procedures to test the significance of the indirect effects. Results indicated that age-diversity climate did not have a significant direct effect on organizational performance. However, the age-diversity climate has an impact on inclusion (β = .61, p < .001) and OCB (β = .13, p < .01). Inclusion showed a positive effect on OCB (β = .41, p < .001) and OCB showed a positive effect on organizational performance (β = .10, p < .05). Additionally, as hypothesized, inclusion and OCB had a mediating role in the relationship between the age-diversity climate and organizational performance (β = .05, s.e. = .02, LLCI = .003, ULCI = .095). The results of this study highlight the importance of the age-diversity climate in the workplace in aging society.

